# Editorial: Role of innate and adaptive immune cells in the metabolic syndrome

**DOI:** 10.3389/fcell.2024.1383642

**Published:** 2024-02-22

**Authors:** Payel Roy, Holger Winkels, Marco Orecchioni, Estefania Quesada-Masachs, Thomas Riffelmacher

**Affiliations:** ^1^ Center for Autoimmunity and Inflammation, La Jolla Institute for Immunology, La Jolla, CA, United States; ^2^ Immunology Center of Georgia, Augusta University, Augusta, GA, United States; ^3^ University of Cologne, Faculty of Medicine and University Hospital Cologne, Clinic III for Internal Medicine, Cologne, Germany; ^4^ Center for Molecular Medicine Cologne (CMMC), University of Cologne, Cologne, Germany; ^5^ Immunometabolism Core Facility, La Jolla Institute for Immunology, La Jolla, CA, United States

**Keywords:** inflammation, metabolism, immune cells, signaling, chronic disease, therapies

Dyslipidemia, hypercholesterolemia, and hyperglycemia are well-recognized metabolic disturbances that control the pathogenesis of a multitude of diseases including obesity-associated metabolic syndrome, diabetes, cardiovascular diseases (CVD), and non-alcoholic fatty liver disease (Hotamisligil, 2017). The presence of lipid-rich and/or glucose-rich microenvironments prompts metabolic adaptations within immune and non-immune cells (Hotamisligil and Erbay, 2008). An intricate crosstalk between the diverse cell types results in the release of a myriad of inflammatory mediators that disrupt crucial metabolic processes including lipolysis, lipogenesis, insulin sensitivity, and glucose utilization (Chawla et al., 2011; Hotamisligil, 2017). This maladaptive inflammation exacerbates progression of several chronic diseases.

In this Research Topic, we curated articles that highlight latest discoveries related to immune cell heterogeneity and crosstalk, activation of signaling pathways, receptor-ligand interactions and release of inflammatory mediators that modulate disease-associated inflammation and metabolic reprogramming.

Mazitova et al. discuss how the complex interplay between fat cells (adipocytes) and myeloid immune cells (macrophages, dendritic cells, neutrophils) regulates the progression of atherosclerosis, a pathological condition that is the most common cause of many CVDs. They describe the various types of adipocytes in the perivascular adipose tissue (PVAT), their unique adipokine profiles, and how these cells in turn alter the immune cell composition and cytokine production in atherosclerotic blood vessels. In this regard, the authors highlight the contributions of Interleukin (IL)-17, Type I and Type II interferons (IFN), IL-6 and IL-27 signaling pathways as important mediators. The authors suggest a dominant role of different functional subtypes of macrophages that are abundantly present both in adipose tissue and in atherosclerotic plaques. Targeting inflammatory responses in a cell-type dependent manner could be a novel therapeutic avenue to address obesity induced meta-inflammation which drives CVD.

Ray and Odum et al. delve deeper into the role of macrophages as regulators of inflammation. They emphasize that diverse macrophage subsets have unique functions in both promoting and resolving inflammation. In this regard, the authors highlight that embryonically-derived resident macrophages and infiltrated monocyte-derived macrophages have distinct and contrasting roles in regulating chronic inflammation. They elaborately review a large body of literature that clarifies our mechanistic understanding of how macrophages exert pro- and anti-inflammatory effects in the context of four chronic diseases - insulin resistance and adipose tissue inflammation, atherosclerosis, non-alcoholic fatty liver disease and neurodegeneration. These factors can be broadly categorized into shared mechanisms related to lipid handling, production of cytokines and lipid mediators, efferocytosis or phagocytosis, and microRNA-mediated gene regulation. These pathways are promising targets for therapy. The authors further cautioned against the blocking of pathways that are active in both pro- and anti-inflammatory macrophage subsets, such as CCR2-CCL2 driven cell recruitment, as this will also impair resolution and tissue repair.

George et al. explore the mechanisms that allow the pleiotropic cytokine, IFN-γ, to both promote and restrain inflammation in the context of type 1 diabetes. As an inflammatory mediator, IFN-γ induces apoptosis and cell death, promotes expression of chemokines, receptors and adhesion molecules that facilitate immune cell recruitment and infiltration, augments antigen presentation and autoreactive T cell activation. On the other hand, IFN-γ has anti-inflammatory effects by limiting the proliferation of pathogenic T cells and by upregulating immune checkpoint molecules in the target tissue. The authors discuss that the pro-inflammatory role of IFN-γ is mediated by the activation of the Janus kinase/signal transducer and activator of the transcription (JAK/STAT) signaling pathway, while the regulatory effect is due to inhibition of JAK/STAT signaling by SOCS1 (suppressors of cytokine signaling 1). Based on this, the authors advocate the use of JAK inhibitors as strategies to limit inflammation in type 1 diabetes.

Liu et al. further extend this possibility and describe the importance of the JAK/STAT signaling pathway in the context of diabetic kidney disease (DKD), the most important microvascular complication of diabetes. The authors extensively reviewed available literature and proposed that JAK/STAT activation can affect the pathogenesis of DKD by influencing multiple factors, including the renin–angiotensin system (RAS), fibrosis, immune cell-mediated inflammation, cellular senescence, autophagy, and epithelial-to-mesenchymal transition (EMT). As these mechanisms are interconnected, inhibition of JAK/STAT signaling can limit disease progression by blocking multiple pathways. Finally, the authors discuss the potential of specific inhibitor molecules, natural compounds and other drugs that target the JAK/STAT pathway, as prospective treatment options in DKD. The safety and efficacy of one of these drugs, Baricitinib, in treating DKD patients, has been explored in a phase II, multicenter, double-blind, randomized, controlled clinical trial.

Guo et al. focus on upstream activators of signaling pathways and shed light on the role of pattern-recognition receptors (PRRs) in orchestrating maladaptive inflammation in endometriosis, a gynecological disorder that is characterized by hormonal imbalance and abnormal growth outside the uterine cavity. Recent studies have shown a pivotal role of immune cells in the etiology of endometriosis. The authors suggest that an inflammatory endometrial microenvironment is maintained by interactions between PRRs on immune cells with pathogen- and damage-associated molecular patterns (PAMPs and DAMPs) in the surrounding milieu. They discuss the potential involvement of all five major groups of PRRs, namely, the toll-like receptors (TLRs), c-type lectin receptors (CLRs), nod-like receptors (NLRs), retinoic acid-inducible gene I-like receptors (RLRs), and absent in melanoma 2 (AIM2)-like receptors (ALRs). The authors propose that PRRs, particularly the TLR molecules, can be targeted to block inflammation.

These studies collectively highlight several distinct and overlapping mechanisms through which immune cells regulate the pathogenesis of inflammatory and metabolic diseases. They emphasize that a complete understanding of diverse pathways needs to be combined with targeted therapies for future clinical success.
